# Classification and substrate head-group specificity of membrane fatty acid desaturases

**DOI:** 10.1016/j.csbj.2016.08.003

**Published:** 2016-09-12

**Authors:** Dongdi Li, Ruth Moorman, Thomas Vanhercke, James Petrie, Surinder Singh, Colin J. Jackson

**Affiliations:** a Research School of Chemistry, Australian National University, Canberra, Australia; b CSIRO Agriculture and Food, Canberra, Australia

**Keywords:** Membrane-bound fatty acid desaturases, Lipid-substrate specificity, Sequence similarity networks (SSNs)

## Abstract

Membrane fatty acid desaturases are a diverse superfamily of enzymes that catalyze the introduction of double bonds into fatty acids. They are essential in a range of metabolic processes, such as the production of omega-3 fatty acids. However, our structure–function understanding of this superfamily is still developing and their range of activities and substrate specificities are broad, and often overlapping, which has made their systematic characterization challenging. A central issue with characterizing these proteins has been the lack of a structural model, which has been overcome with the recent publication of the crystal structures of two mammalian fatty acid desaturases. In this work, we have used sequence similarity networks to investigate the similarity among over 5000 related membrane fatty acid desaturase sequences, leading to a detailed classification of the superfamily, families and subfamilies with regard to their function and substrate head-group specificity. This work will facilitate rapid prediction of the function and specificity of new and existing sequences, as well as forming a basis for future efforts to manipulate the substrate specificity of these proteins for biotechnology applications.

## 1 Introduction

**Fig. 1:**
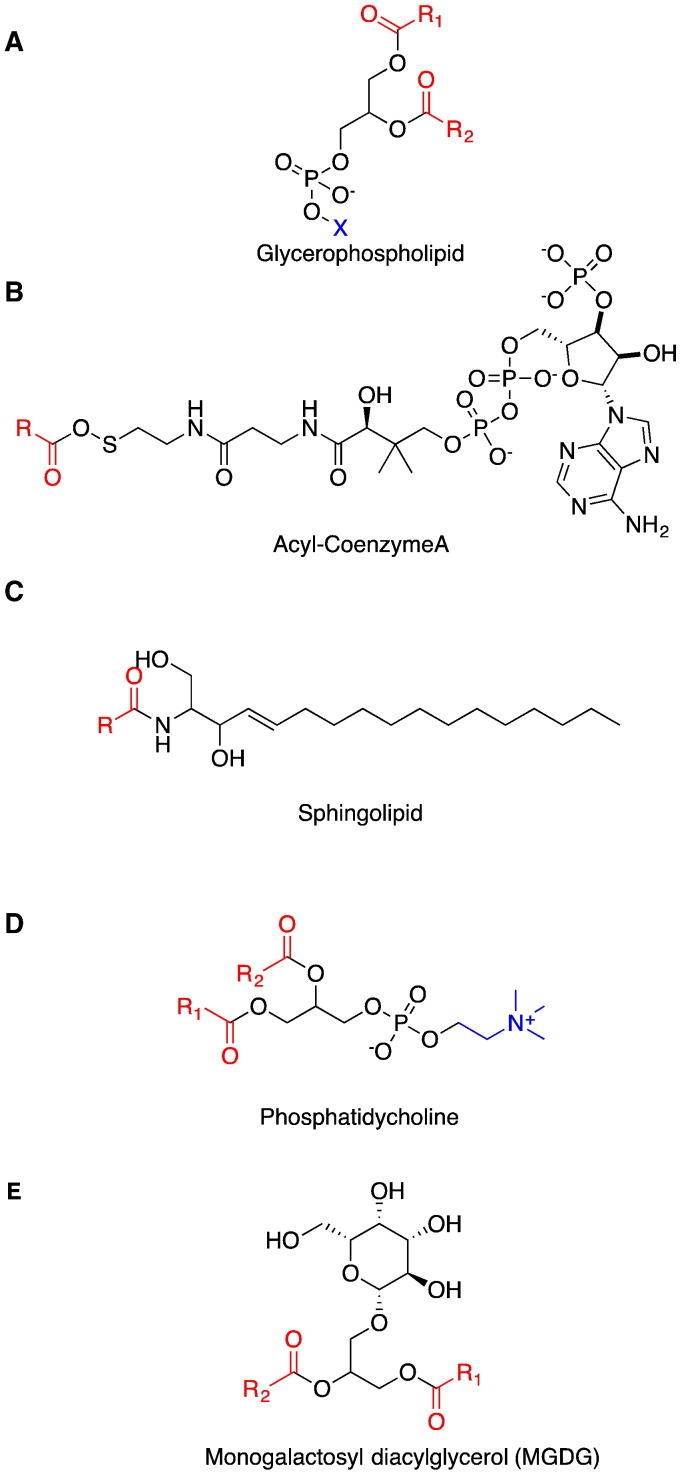
The chemical structures of glycerophospholipid (A), acyl-Coenzyme A (B), sphingolipid (SP), phosphatidycholine as a major phospholipid (PC) and monogalactosyldiacylglycerol (MGDG). The acyl groups are coloured red. The X head-group on the glycerophospholipid backbone varies between different classes with the structure of PC given as an example.

In contrast to soluble fatty acid desaturases (FADs), which are acyl–acyl carrier protein (acyl-ACP) specific [1], [2], membrane FADs are a diverse family of proteins that display a range of lipid substrate preferences including acyl-CoAs, sphingolipids (SP), phospholipids (PL) and galactolipids (GL) [3], [4]. The acyl chains of these substrates can, in general, be quite similar, but the “head-groups” of these lipids differ and contribute to the different physiological roles (Fig. 1). For example, acyl-ACPs are important intermediates for a number of different metabolic pathways, including lipid biosynthesis [5], whereas PL and GL are not only important structural lipids, but also play essential roles in different cellular signalling pathways [6]. Monogalactosyl diacylglycerol (MGDG) is a type of non-phosphorous structural glycerolipid that is abundant in the cellular membranes of photosynthetic organisms [7], [8], [9]. Similarly, SP is also a class of structural lipids, although its roles in the regulation of cellular processes, such as apoptosis, cell migration and cold tolerance, makes this class of lipid particularly important [10], [11], [12], [13]. Acyl-CoA substrates are frequently acted upon by lipid-modifying enzymes such as desaturases, acyltransferases and elongases [14], [15], [16], [17], [18], [19]. Therefore, acyl-CoA substrates are especially important for the production of unusual lipids such as docosahexaenoic acid and eicosapentaenoic acid [14], [20].

The substrate specificity and regioselectivity (double bond positioning) of membrane FADs is largely determined by the interaction between the enzyme and the lipid head-group. The recently published human and mouse steaoryl-CoA desaturase structures reveal the interaction between the lipid substrate and the membrane FADs [21], [22]. The hydrophilic CoA head-group of the substrate forms electrostatic interactions and hydrogen bonds with residues in the cytoplasmic domain and transmembrane helix (TM) 1 of the desaturase, and orients the acyl group into the long hydrophobic tunnel with the target carbon presented at di-metal active site [22]. The acyl group is surrounded by hydrophobic residues in the substrate binding cavity including W262 on TM4, which holds the substrate in place for Δ9 desaturation [22]. However, the substrate binding mechanisms of the desaturases that are not specific to acyl-CoA are still poorly understood. A domain-swapping study between the acyl-phosphatidylcholine (acyl-PC) specific Δ6 desaturase and the sphingolipid specific Δ8 desaturase from *Borage officinalis* discovered that TM helices, as well as the cytosolic loops, of the desaturases play essential roles in differentiating between head-groups [23]. However, there is not enough structural data to either identify specific roles for the functionally important residues in substrate binding, or to describe an accurate mechanism for the substrate binding of the acyl-PC-specific and sphingolipid-specific FADs. Moreover, there is currently no established sequence-based classification available that accurately predicts head-group specificity and regioselectivity of uncharacterized sequences.

Based on their regiospecificity and sequence homology, the membrane desaturase superfamily has been further divided into families [3], [24], [25]. Front-end (FE) desaturases are capable of introducing carbon–carbon double bonds into unsaturated lipid substrates at positions between an existing double bond and the terminal carboxyl group [4]. It was found that the varied substrate preference of the front-end desaturases is the root cause of the substrate dichotomy bottleneck in the ω3-LCPUFAs biosynthesis pathway [20], [63], [64]. This is because some Δ6 desaturases utilize acyl-PC substrates, whereas the elongases at the next step utilize acyl-CoA substrates [20]. Thus, for acyl-PC-specific Δ6 desaturases, the product of the Δ6 desaturation has to be converted into an acyl-CoA molecule by an acyltransferase, which limits the metabolic flux in the recombinant ω3-LCPUFA biosynthesis pathway in plants [20]. Second, first desaturases (FDs), typically catalyze the formation of the first C = C bond at the Δ9 position of an acyl group. It has been postulated that the Δ9 desaturases are the most ancient desaturases among the three groups because of their universal distribution in organisms [24]. The FDs utilize acyl-CoA as substrates except for the FDs of plant plastids [8]. Third, the methyl-end (ME) desaturases are responsible for introducing a C=C between a pre-existing C=C and the methyl end of the acyl group [26]. ME desaturases are known to utilize PL substrates [27], [28]. Finally, the Δ4 sphingolipid desaturases (Δ4-SPs) are particularly important for cellular signalling [29]. This classification was based on a previous limited phylogenetic analysis of eukaryotic membrane-bound desaturases [25].

For a very diverse protein family, such as the membrane FADs, constructing a high-quality multiple sequence alignment can be challenging [30], which limits our ability to obtain evolutionary information from phylogenetic analysis and to annotate the possible functions for identified genes with confidence [30]. Protein sequence similarity networks (SSNs) were developed in 2009 by Babbitt and co-workers to facilitate functional annotation based on known sequence data [31]. SSNs can illustrate the global sequence–structure–function diversity of protein superfamilies [32], [33], [34], because they are based on many pairwise alignments of proteins instead of the entire alignment of a large protein datasets, so that the inaccuracy of a large protein alignment is largely eliminated. A SSN presents the level of similarity between members in a protein family in a graphical way in which proteins sharing high sequence identities are clustered. In the SSNs, the edges represent all*-vs-*all BLAST *E*-values and each node represents a protein or a group of highly similar proteins. The information about their organism of origin and structural understanding can be mapped and visualized in the network manually or automatically [35]. This method can be combined with other computational or biochemical techniques to annotate and characterize the function–structure relationships of protein subfamilies. Two example applications of the use of SSNs are the re-classifications of the cytosolic glutathione transferases superfamily [36], and an oxidoreductase superfamily [30], where biochemical characterization is incorporated to provide a more in-depth analysis of the functional and structural diversity of protein subfamilies. Thus, SSNs can serve as the foundation for the characterization of protein superfamilies by providing information about the sequence–structure–function divergence [37].

In this work, membrane FADs were analysed using SSNs to determine the distinctions in sequence and structure to provide a clearer picture of the differences in the possible mechanisms of substrate binding. Because of the high commercial value of the FE desaturases in the production of poly-unsaturated fatty acids, such as ω3-LCPUFAs [14], a more in-depth analysis of this family was performed. These results provide new directions for the future engineering membrane FADs.

## 2 Results and discussion

### 2.1 The sequence–structure–function relationships of all known membrane-bound desaturases

**Fig. 2:**
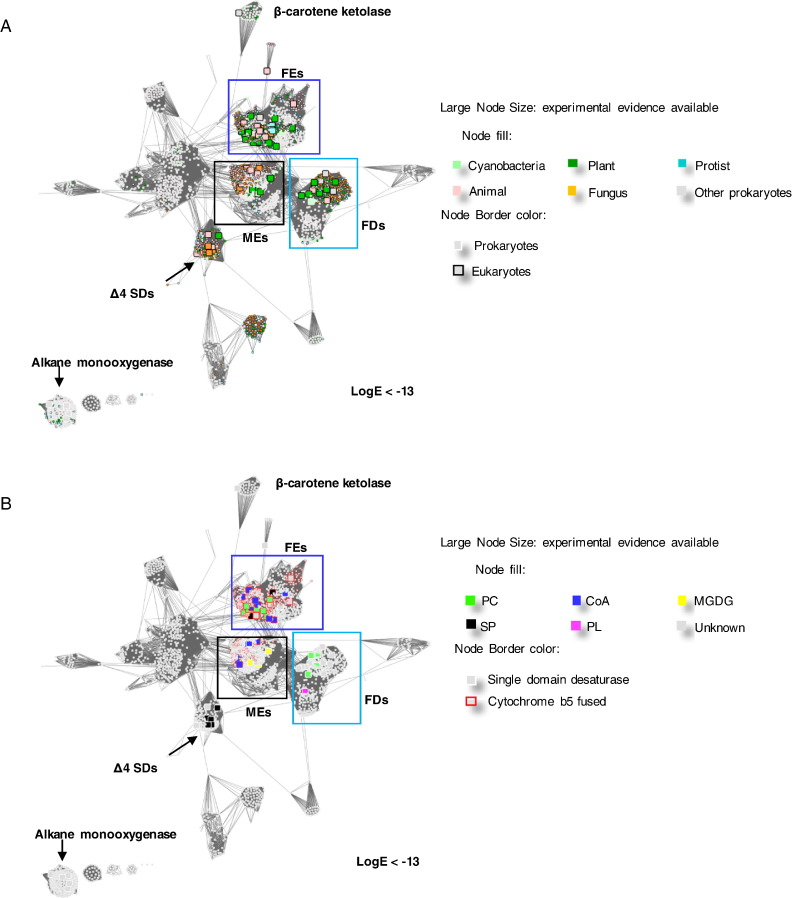
Overview of the sequence similarity relationships in the membrane-bound desaturase family. These representative networks show 2878 nodes representing the 5245 proteins in the membrane-bound desaturase family, in the Pfam database (pfam.xfam.org) (the list of the UniProt IDs of the sequences included in this network are provided in Supplementary Table S1). The clusters with functionally characterized member sequences are labelled, including the functionally dissimilar alkane monooxygenases cluster and beta-carotene ketolases clusters. There are three major fatty acid desaturase families (first desaturases, FDs; methyl-end desaturases, MEs; front-end desaturases, FEs). Δ4 sphingolipid desaturases (SDs) are also shown. Larger squares represent nodes with at least one functionally characterized member. Edges or lines connecting the nodes are shown if the pairwise similarity score between the sequences of representative nodes is lower than the threshold of Log BLAST E-value of − 13. The 366,864 edges in the overview network have a median sequence identity of 30% over 275 residues. A and B are identical networks with different colour coding as explained in the figure keys. A. The nodes in this overview network are coloured by the kingdom of the organism of origin. B. The nodes in this overview network are coloured by the substrate head-group specificity of the characterized members. Overview of the sequence similarity relationships in the membrane-bound desaturase family. These representative networks show 2878 nodes representing the 5245 proteins in the membrane-bound desaturase family, in the Pfam database (pfam.xfam.org) (the list of the UniProt IDs of the sequences included in this network are provided in Supplementary Table S1). The clusters with functionally characterized member sequences are labelled, including the functionally dissimilar alkane monooxygenases cluster and beta-carotene ketolases clusters. There are three major fatty acid desaturase families (first desaturases, FDs; methyl-end desaturases, MEs; front-end desaturases, FEs). Δ4 sphingolipid desaturases (SDs) are also shown. Larger squares represent nodes with at least one functionally characterized member. Edges or lines connecting the nodes are shown if the pairwise similarity score between the sequences of representative nodes is lower than the threshold of Log BLAST E-value of − 13. The 366,864 edges in the overview network have a median sequence identity of 30% over 275 residues. A and B are identical networks with different colour coding as explained in the figure keys. A. The nodes in this overview network are coloured by the kingdom of the organism of origin. B. The nodes in this overview network are coloured by the substrate head-group specificity of the characterized members.

In order to characterize the sequence and functional diversity in the membrane FADs, particularly the substrate head-group specificity, 5245 sequences were collected using the PFAM fatty acid desaturase family PF00487 as seed sequence clusters within the length range of 350 aa to 550 aa. This length range was chosen to limit the search to the single domain desaturases and the desaturases with a fused cytochrome b5 domain. The free cytochrome b5 proteins and the unrelated long cytochrome b5 domain containing fusion proteins are eliminated with this filter. The collected sequences were analysed by generating a SSN using EFI-EST [35], where an initial network containing 2878 representative nodes (clusters of protein sequences with > 60% amino acid identity) was produced. The edges represent all-*vs*-all BLAST *E*-values between the clusters. The cytochrome b5 domain was only included in the *E*-value calculations when all of the protein members in the connecting nodes also contained cytochrome b5 domains. Otherwise, only the fatty acid desaturase sequences were used to calculate the *E*-values. A literature review was performed to collect the known lipid head-group preferences of the characterized desaturases and their organism distributions. This data was mapped onto the networks (Fig. 2).

The SSNs in Fig. 2 are presented with different colour coding of the nodes to identify the kingdom in which the respective membrane FADs are found and the substrate head-group preference. It is apparent that there are a large number of uncharacterized prokaryotic desaturase-like proteins in the database. The functionally distinct beta-carotene ketolases and alkane monooxygenases are also present in the network, because they all carry the three conserved and functionally important histidine-rich motifs, which coordinate the catalytic di-iron centres of these enzymes [38], [39], [40]. However, they are sufficiently different in terms of sequence identity and function to be excluded from this membrane FAD-focussed study. Four major clusters of desaturases with characterized members are visualized in the SSNs. By examining the characterized members in each major cluster, the four clusters are identified as the first desaturases (FDs) [41], [42], methyl-end desaturases (MEs) [43], front-end desaturases (FEs) [4] and Δ4 sphingolipid desaturases (Δ4SPs) [44]. The different clusters include multiple sequences from different evolutionary kingdoms as well as a range of different substrate preferences (Fig. 2). This coarse separation of the clusters is consistent with the previously proposed membrane FAD classification, which was based on the phylogenetic analysis of eukaryotic membrane-bound desaturases [25]. The Δ4 sphingolipid desaturases catalyze the Δ4 desaturation of important signalling molecules such as (E)-sphing-4-enine-1-phosphate, which is the messenger for the epidermal growth factor (EGF) receptor family [29]. As Δ4s are not known to be specific for substrates other than sphingolipids, they were not subjected to further detailed analysis in this study.

### 2.2 Substrate specificity in the first desaturase family is determined by the presence of charged residues within the substrate binding site

**Fig. 3:**
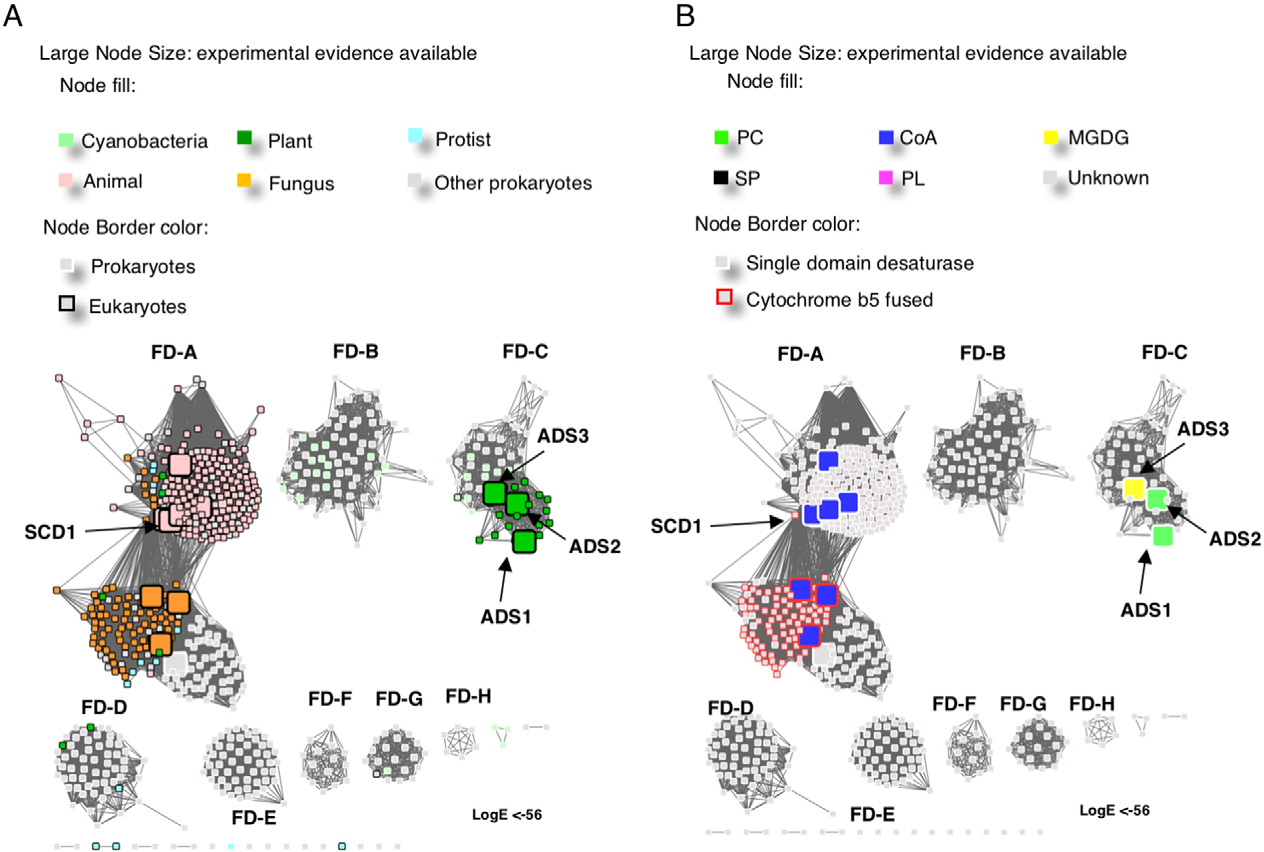
The representative networks of the FDs show more detailed subgroupings. A and B are the same networks generated from the FDs cluster in Fig. 2 at a higher stringency, LogE < − 56. Eight major clusters (clusters with more than 50 member sequences in each) formed. The nodes are coloured by organism kingdom information (A) or their substrate specificities (B).

The three main families within the membrane FAD superfamily (FD, ME, FE) can be further resolved by reducing the LogE filter to remove weak associations. The FD family contains the structurally characterized mammalian Δ9 stearoyl-CoA desaturases (SCD1s) [41], which share approximately 32% amino acid identity with other members of the FD subfamily. They are responsible for introducing the first C=C bond into a saturated hydrocarbon chain [21]. At a logE value of − 56, eight major clusters are formed (Fig. 3). The largest cluster consists of acyl-CoA specific Δ9 FDs (FD-A). The only other cluster that contains experimentally characterized desaturases is the FD-C cluster, which contain the characterized bi-functional acyl-lipid-specific *Arabidopsis thaliana* Δ7/Δ9 desaturases (ADS1) [8].

The FD-A cluster consists of the acyl-CoA Δ9 single domain FADs, predominantly from animals, and fusion desaturases that have a cytochrome b5 domain fused at the C-terminus of the desaturase, predominantly from fungi (Fig. 3). Two crystal structures of single-domain animal desaturases have been published and have defined the geometry of the acyl-CoA as bound in the desaturase [21], [22]. However, there is no crystal structure of the fused fungal desaturases to date for structural comparison. Given that there is significant amino acid sequence identity (higher than 30%) in the desaturase domain between the fused and unfused proteins, the former most likely exhibit similar binding characteristics as the single domain desaturases.

The FD-C cluster is composed of plant plastid FADs and prokaryotic FADs, including the bi-functional ADS1 protein, which revealed an intriguing subcellular-specific substrate-specificity and regioselectivity [8]. This desaturase is capable of catalysing Δ9 desaturation of acyl-PC, when targeted to endoplasmic reticulum (ER) membrane [8]. It can also catalyze Δ7 desaturation of acyl-groups presented on MGDG when targeted to the plastid membrane [8]. However, because most ADS enzymes (with the exception of the plastid-localized ADS3) are localized to the ER, their native function is likely to involve desaturation of acyl-PC. As the structures of MGDG and acyl-PC are only different by the hydrophilic functional head-groups at the sn3 position of the glycerol backbone (Fig. 1), overlapping substrate specificity is not entirely surprising.

**Fig. 4:**
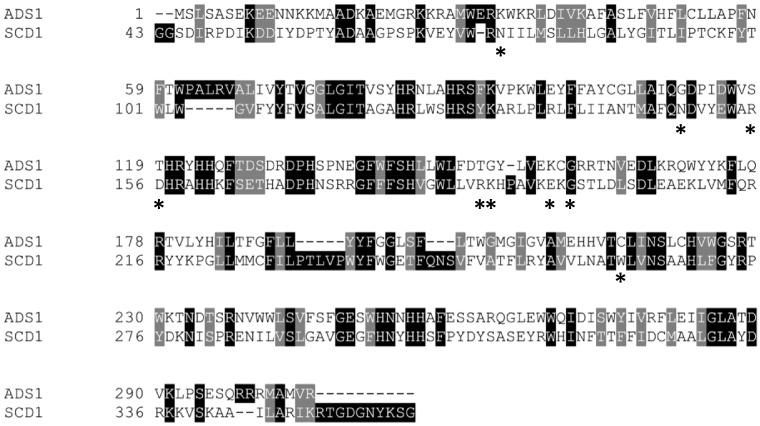
The alignment of ADS1 and SCD1. The substrate head-group binding residues of the crystal structure of SCD1 [22] are denoted by asterisks.

**Fig. 5:**
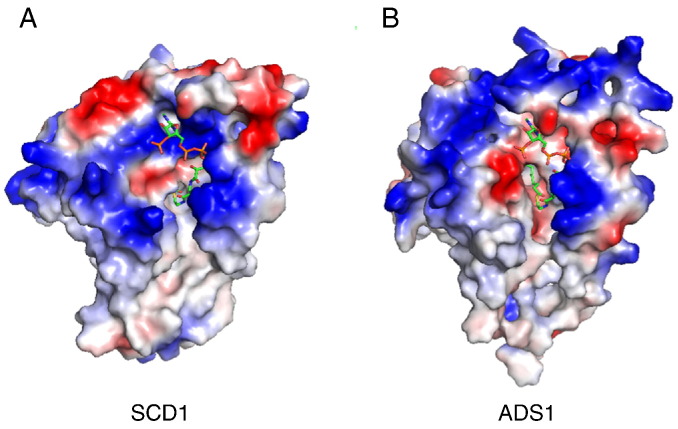
The surface charge distribution comparison between an acyl-CoA-specific FD-A and an MGDG-specific FD-C. The difference in surface charge distributions visualized on the surface of the crystal structure of human steaoryl-CoA Δ9 desaturase (SCD1, PDB ID: 4ZYO) and the homology model of ADS1 inside and around the substrate-binding cavity.

Although both FD-A and FD-C clusters belong to the FD family, they have diverged in terms of head-group specificity. When the sequence differences between the FD-A human SCD1 and the FD-C ADS1 and are compared, a number of non-conservative substitutions are evident (Fig. 4). Specifically, a number of charged or polar residues that are responsible for CoA binding in SCD1 are replaced by uncharged amino acids in ADS1, which result in significant changes in the surface charge distribution in the substrate binding cavity (Fig. 5). The loss of positive charge, which is complementary to the extensive negative charge on coenzyme-A but is not required for binding of the glycerol-sugar moiety of MGDG, is consistent with the change in specificity.

### 2.3 Methyl-end desaturases specific for phospholipid substrates

**Fig. 6:**
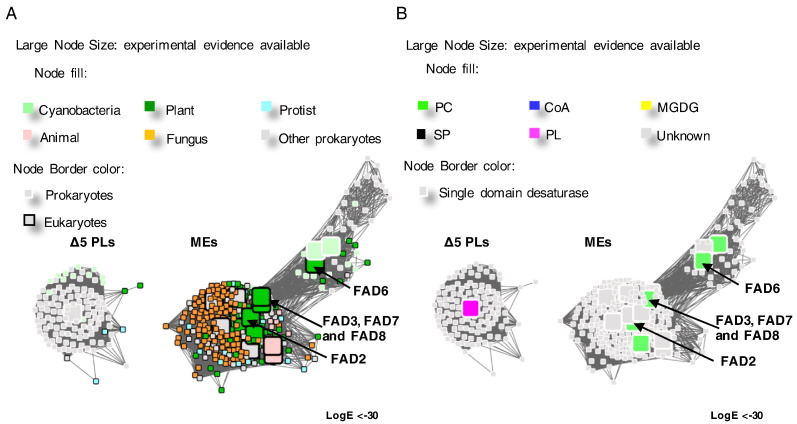
The representative network of the MEs cluster in Fig. 2 show more detailed subgroupings. The plant FAD2, FAD3, FAD6, FAD7 and FAD8 desaturases are denoted. A and B are the same networks generated from the MEs cluster in Fig. 2 at a higher stringency, LogE < − 56. The prokaryotic Δ5 PLs formed a separate cluster. The nodes are coloured by organism kingdom information (A) and their substrate specificities reported in the literature (B).

The methyl-end desaturase subfamily can be separated into two groups at the LogE filter of − 30: a prokaryotic Δ5 phospholipid FAD group (Δ5 PLs) and the canonical MEs including Δ12-specific [45], Δ15-specific [46] and bifunctional (Δ12 and Δ15) FADs [47] (Fig. 6). The Δ5 PL desaturase from *Bacillus subtilis* (UniProt ID: O34653) has been experimentally proven to have a six-transmembrane (TM) helix topology [27], [28]. Functionally, the Δ5 PL cluster is similar to the first desaturases, although it shares higher sequence similarity with the ME family. Given the high amino acid sequence identity between these clusters (higher than 21%), the 6-TM-helix topology is likely to be the common topology of the MEs cluster and makes this subfamily structurally distinct from the FDs. Even though the bacterial *B. subtilis* Δ5 desaturase is suggested to be specific for phospholipid, this study did not specify the class of phospholipid that is the primary substrate [27], [28]. Several enzymes from the ME cluster have been shown to catalyze the desaturation of phosphatidylcholine including the plant FAD2 FAD3, FAD6, FAD7 and FAD8 desaturases (Fig. 6) [48], [49], [50], [51]. Thus, it is likely that both clusters utilize phospholipid substrates. It is notable that the Δ5 phospholipid FAD group consists almost entirely of prokaryotic and cyanobacterial sequences (with a handful of sequences from plants and protists), whereas the canonical ME cluster consists of sequences from fungi, animals, plants, cyanobacteria and other prokaryotes.

### 2.4 Front-end desaturases have diverse substrate specificity

**Fig. 7:**
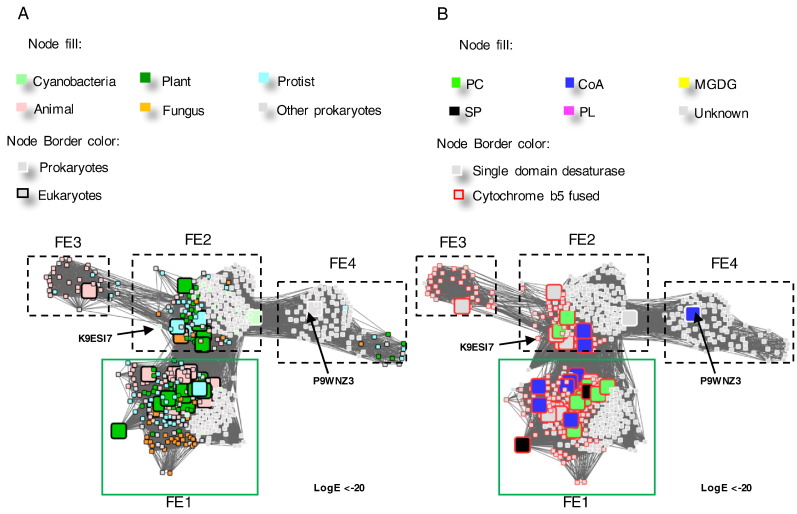
Separation of the FE desaturase family into smaller groups. A and B are the same networks generated from the FEs cluster in Fig. 2 at a higher stringency, LogE < − 20. The nodes are coloured by organism kingdom information (A) and their substrate specificities reported in the literature (B).

The FE desaturases are responsible for introducing carbon–carbon double bonds into unsaturated acyl chains between the pre-existing double bonds and the carboxyl group of lipid substrates [52]. There is currently no crystal structure of any member of the FE desaturase family. Thus, our understanding of their mechanism and regioselectivity is primarily based on mutagenesis studies, which have been reviewed by Meesapyodsuk and Qiu [4]. A range of FEs with Δ4, Δ5, Δ6 desaturases activity and specificity for acyl-CoA or acyl-PC substrates have been identified [4], [20], [53], [54], [55], [56], [57]. This level of diversity is not seen in the other FAD families, making FE desaturases distinct from MEs and FDs. With a LogE < − 20 filter, the broad FE desaturase family separated into four clusters. For ease of explanation, the clades are named as FE1–4 (Fig. 7). The fusion-FADs, which have a cytochrome b5 domain fused at the N-terminus of the desaturase domain, are only found in the FE1–3 clusters.

Clusters 1 and 2 of the FE desaturases are the largest and both include sequences from prokaryotes and eukaryotes, although only the eukaryotic proteins have fused cytochrome b5 domains at the N-termini. This observation suggests that the gene fusion event likely took place after the evolution of eukaryotes. The FE1 cluster includes the Δ6 desaturases from *M. pusilla* and *O. tauri*, which have been shown to function as desaturases with omega-3 and omega-6 fatty acids [14], [58]. A single prokaryotic fusion desaturase from the cyanobacterium *Leptolyngbya* sp. PCC 7375 (UniProt ID: K9ESI7) falls within those found in the FE2 cluster. It is unclear if K9ESI7 is the result of a horizontal gene transfer event between eukaryotes and prokaryotes, or if it is the descendent of an ancient gene fusion that was the evolutionary origin of the eukaryotic fusion proteins. The smallest cluster (FE3) includes proteins that are predominantly found in insects, with the exception of a few proteins from algae (*Nannochloropsis gaditana*) and simple eukaryotes and are believed to be housekeeping genes involved in lipid metabolism [59]. Finally, the FE4 cluster is primarily composed of single domain (non-fused) desaturases from prokaryotic species, but also includes some genes from eukaryotes that encode single domain proteins. The only characterized protein in this family is the NADPH oxidoreductase-dependent C16:0/C18:0-CoA Δ9 desaturase from *Mycobacterium* (UniProt ID: P9WNZ3) [60]. Its function mimics the role of FDs as this enzyme is responsible for introducing the first double bond into fatty acid chains.

**Fig. 8:**
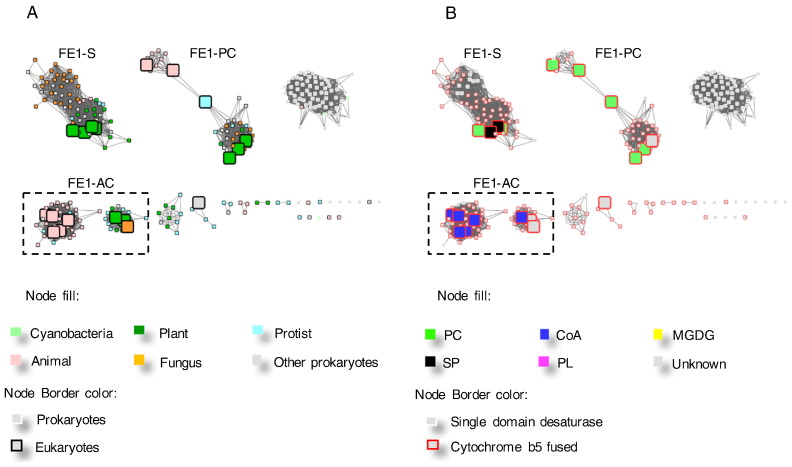
The representative sub-clusters within the FE1 cluster. A and B are the same networks generated from the FE1 cluster in Fig. 2 at a higher stringency, LogE < − 65. The nodes are coloured by organism kingdom information (A) and their substrate specificities reported in the literature (B).

**Fig. 9:**
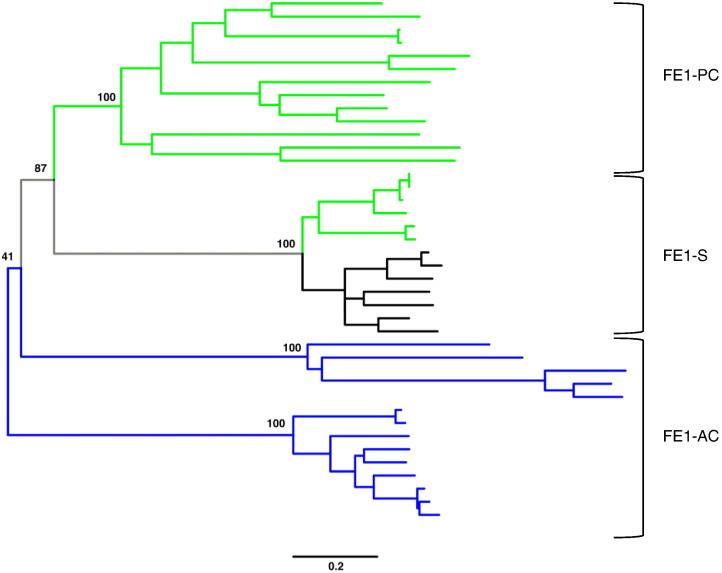
Phylogenetic analysis of the FE1 sub-cluster. The tree was constructed using MEGA v.7. A list of the included desaturase proteins is detailed in the Supplementary Materials. The bootstrap values of 100 replicates are denoted at the major nodes. The green branches indicate acyl-PC specificity. The black branches indicate sphingolipid specificity. The blue branches indicate acyl-CoA specificity.

Increasing the LogE filter to <− 65 can further differentiate the FE1 cluster, revealing the presence of clusters with distinct lipid head-group preferences including a sphingolipids/acyl-PC-specific cluster (FE1-S), a predominantly acyl-PC-specific cluster (FE1-PC) and acyl-CoA-specific proteins (FE1-AC) (Fig. 8). The separation of sequences within the FE1 sub-cluster is supported by a phylogenetic analysis of the functionally characterized FE desaturases (Fig. 9). The FE1-AC sub-cluster includes the animal Δ5 and Δ6 acyl-CoA desaturases, as well as the algal and fungal acyl-CoA desaturases. The FE1-PC sub-cluster includes genes from *C. elegans*, moss and liverworts. Within this sub-cluster, *Marchantia polymorpha* Δ6 desaturase (UniProt ID: Q696V8) has been shown to have some promiscuous activity with acyl-CoA substrates in addition to acyl-PC substrates [61]. The FE1-S sub-cluster is complex and difficult to resolve at a LogE < − 65 filter, and includes both acyl-PC and acyl-SP desaturases. Within the FE1-S sub-cluster, higher plant FE desaturases cluster with a group of fungal proteins.

The topology of the FE cluster has not been experimentally confirmed, which makes it difficult to determine the specific substrate-binding mode of these desaturases. It is possible that the modes of interaction between the proteins of the FE cluster and the different types of lipid substrates could also be distinctive from the observed binding mechanism in SCD1 [22]. The recently published structure of the yeast integral membrane fatty acid α-hydroxylase (scScs7p) [62] could indicate an alternative lipid–substrate binding mechanism that might be relevant to these enzymes. However, given that it only shares very low (~ 14%) amino acid sequence identity with members of the FE cluster, we think that the structures are likely to be too different to allow the inference of alternative substrate-binding modes.

## 3 Conclusion

This broad sequence–structure survey of the membrane FAD superfamily provides several notable observations. First, there are a large number of uncharacterized FADs that are essentially distinct from all known clusters and these proteins are abundant in prokaryotes. Further work to characterize these abundant proteins will be necessary to identify their evidently important physiological roles. Second, the membrane FAD superfamily is diverse, with the divergence of FE, ME, FD families, as well as a number of smaller and more specialized families, such as the Δ4 sphingolipid desaturases. This evolutionary separation of proteins on the basis of their function and regioselectivity (first, methyl end, or front end) produces a number of sub-clusters in the FD and FE families that have specificity for fatty acids with different head-groups. The FE desaturases, for example, have evolved to become specific for acyl-CoA, acyl-PC and sphingolipid substrates. In this work, we have classified this diverse superfamily for the first time in detail. This figure also highlights the gaps in our current understanding—for instance, what are the functions of the FD2 and FD4 groups?

## 4 Materials and methods

### 4.1 Sequence similarity networks (SSNs)

SSNs were generated by EFI-EFT using sequences belonging to the PF00478 fatty acid desaturase superfamily in PFAM database, in which the BLAST *all-vs-all* LogE values were used as the edges and a LogE cutoff of − 5 was applied to the initial network generation [35], [63]. The network consists of the nodes representing protein clusters with 60% sequence identity which were visualized using Cytoscape v.3.2.1 [64]. The sequences shorter than 250 aa or longer than 550 aa were excluded to avoid partial proteins or proteins with more than two domains. The network was curated to remove unrelated sequences. The subgroupings in each major cluster were visualized by gradually increasing the stringency of the LogE filter of the networks. The published functional data was used to determine the consensus function and substrate preference of each subfamily.

### 4.2 Phylogenetic analysis

The FE desaturases with experimentally proven functional data were collected from the literature. A total of 38 sequences (Supplementary Table S2) belonging to FE1 cluster were aligned by Molecular Evolutionary Genetics Analysis program Version 7 (MEGA7) using MUSCLE algorithm [65], [66]. A phylogenetic tree was constructed using the maximum-likelihood method and LG matrix in (MEGA7) [65].

### 4.3 Structural modelling

The protein sequence of the *Arabidopsis thaliana* Δ9 desaturase (ADS1, UniProt ID: O65797) was submitted to the Phyre2 modelling server [67] using the default settings of the "normal" modelling mode, with human stearoyl-CoA desaturase (PDB: 4ZYO) as template.

The following are the supplementary data related to this article.

Supplementary Table S1 The UniProt IDs of sequences included in Fig. 2 Supplementary Table S1

Supplementary Table S2 Sequences of the characterized members of FE1 sub-cluster used to generate the phylogenetic tree in Fig. 9. Supplementary Table S2
