# Supplementary material for: Classification and substrate head-group specificity of membrane fatty acid desaturases
Source: Comput Struct Biotechnol J. 2016 Sep 12;14:341–9. doi: 10.1016/j.csbj.2016.08.003 (PMC5037126; doi:10.1016/j.csbj.2016.08.003)
Supplement: Supplementary Table S2 — Sequences of the characterized members of FE1 sub-cluster used to generate the phylogenetic tree in Fig. 9. [file mmc2.docx]

**Supplementary Table S2.** Sequences of the characterized members of FE1 sub-cluster used to generate the phylogenetic tree in Figure 9.

| Organism | UniProt ID |
| --- | --- |
| Rhizopus oryzae | Q6PS62 |
| Amylomyces rouxii | Q6TMX2 |
| Mortierella isabellina | Q8X173 |
| Mortierella alpina | Q9UVV3 |
| Thalassiosira pseudonana | Q4G2T1 |
| Phaeodactylum tricornutum | Q8RXB0 |
| Marchantia polymorpha | Q696V8 |
| Ceratodon purpureus | Q9LEN0 |
| Ceratodon purpureus | Q9LEM9 |
| Physcomitrella patens subsp. patens | Q9ZNW2 |
| Caenorhabditis elegans | G5EG11 |
| Caenorhabditis elegans | Q23221 |
| Echium plantagineum | Q2M488 |
| Euglena gracilis | Q8VZZ2 |
| Borago officinalis | O04353 |
| Primula farinosa | Q84KG8 |
| Primula vialii | Q84KG6 |
| Brassica rapa | G9I7G1 |
| Brassica rapa | D2KBG5 |
| Brassica rapa | D2KBG3 |
| Helianthus annuus | Q43469 |
| Borago officinalis | Q9FR82 |
| Arabidopsis thaliana | Q9ZRP7 |
| Arabidopsis thaliana | Q3EBF7 |
| Thraustochytrium sp. | Q8S3C1 |
| Rebecca salina | A4KDP0 |
| Ostreococcus tauri | Q4JDG7 |
| Ostreococcus lucimarinus CCE9901 | G2J5R1 |
| Micromonas pusilla (strain CCMP1545) | C1MMV2 |
| Mus musculus | Q920L1 |
| Danio rerio | Q9DEX7 |
| Sparus aurata | Q8AY64 |
| Rattus norvegicus | Q9Z122 |
| Rattus norvegicus | Q920R3 |
| Salmo salar | Q6SES0 |
| Scophthalmus maximus | Q6QDP0 |
| Oncorhynchus mykiss | Q98SW7 |
| Salmo salar | Q8QGE2 |
